# Progress and Hurdles of Therapeutic Nanosystems against Cancer

**DOI:** 10.3390/pharmaceutics14020388

**Published:** 2022-02-10

**Authors:** Marina Martín-Contreras, Saúl A. Navarro-Marchal, José Manuel Peula-García, Ana Belén Jódar-Reyes

**Affiliations:** 1 Department of Applied Physics, University of Granada, Campus Fuentenueva s/n, 18071 Granada, Spain; marinamctr.97@gmail.com (M.M.-C.); navarrosa@ugr.es (S.A.N.-M.); 2 Centre for Biomedical Research (CIBM), Biopathology and Regenerative Medicine Institute (IBIMER), University of Granada, 18100 Granada, Spain; 3 Instituto de Investigación Biosanitaria (ibs.GRANADA), University Hospitals of Granada, University of Granada, 18100 Granada, Spain; 4 Department of Chemistry in Pharmaceutical Sciences, Faculty of Pharmacy, Complutense University of Madrid, Plaza Ramón y Cajal s/n, 28040 Madrid, Spain; 5 Excellence Research Unit Modeling Nature (MNat), University of Granada, 18071 Granada, Spain; 6 Department of Applied Physics II, University of Málaga, 29016 Malaga, Spain; jmpeula@uma.es; 7 Biocolloid and Fluid Physics Group, University of Granada, 18071 Granada, Spain

**Keywords:** nanomedicine, design of nanosystems, physicochemical properties, protein corona, targeting, delivery, clinical application

## Abstract

Nanomedicine against cancer, including diagnosis, prevention and treatment, has increased expectations for the solution of many biomedical challenges in the fight against this disease. In recent decades, an exhaustive design of nanosystems with high specificity, sensitivity and selectivity has been achieved due to a rigorous control over their physicochemical properties and an understanding of the nano–bio interface. However, despite the considerable progress that has been reached in this field, there are still different hurdles that limit the clinical application of these nanosystems, which, along with their possible solutions, have been reviewed in this work. Specifically, physiological processes as biological barriers and protein corona formation related to the administration routes, designing strategies to overcome these obstacles, promising new multifunctional nanotherapeutics, and recent clinical trials are presented in this review.

## 1. Introduction

Cancer is a group of diseases characterized by an abnormal growth of tumour cells with the ability to invade other areas of the body. More than 10 million new cases are diagnosed annually, and the World Health Organization (WHO) estimates that cancer-related deaths will rise to approximately 13.1 million by 2030. Therefore, this is one of the most devastating diseases in the world [1].

**Figure 1:**
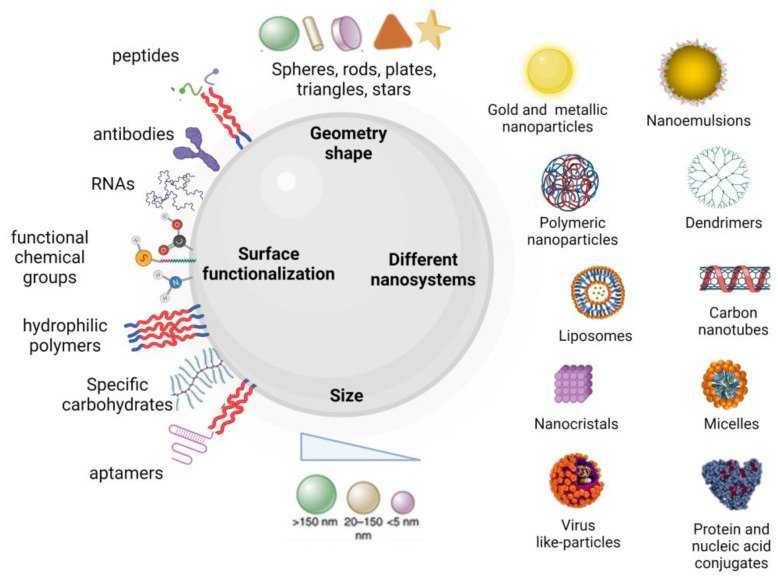
Schematic representation of the usual size, morphology, surface chemistry and types of nanomaterials employed in cancer therapy.

Conventional cancer therapies have intrinsic limits, such as the low water solubility of a large number of anti-tumour drugs, the high non-specificity and the emergence of multi-drug resistance (MDR) after repeated administration, which restrict their therapeutic efficacy. These limitations prompted the development and application of several biomedical technologies, such as nanomedicine, which is considered to be the medical use of nanotechnology for the diagnosis, prevention and treatment of diseases through the understanding of the biochemical, physicochemical and physiological processes involving a given pathology [2,3,4]. This description encompasses three nanotechnology areas: (1) drug delivery, consisting of the design of nanostructured biomaterials that transport and deliver therapeutic loads to the target site in a controlled manner; (2) diagnosis, focusing on the development of imaging nanosystems or nanobiosensors that identify a given pathology at a cellular or molecular level with high sensitivity; and (3) theranostics, combining the design and application of nanomaterials to determine a specific pathology, and simultaneous drug delivery. In general, the active molecules are incorporated into the nanosystems through a variety of mechanisms, including physical encapsulation, adsorption, chemical conjugation, or a combination of these. They are delivered to the body and reach the tumour site in a specific and efficient way [3]. The “nano” world offers exclusive properties for cancer treatment, due to the possibility of designing different nanosystems (inorganic nanoparticles, dendrimers, proteins conjugated with the active molecule, polymer micelles, liposomes, carbon nanotubes (CNT), quantum dots (QD), biopolymer nanoparticles and their combinations) with particular intrinsic properties for each. Moreover, they can be synthesized from different materials to acquire diverse physicochemical properties [5] (i.e., morphology, surface chemistry, size and consequently, different surface area, Figure 1). Due to this possibility of a comprehensive design, there are numerous benefits that can be obtained from multifunctional nanosystems in terms of the ability to diagnose and treat cancer earlier and more effectively [1,2].

The aim of nanomedicine against cancer is to improve the therapeutic windows of the different treatments [6,7]. The qualities of the nanoplatforms would allow: (1) Improved absorption and stability of low water-soluble drugs; (2) Reduction in the systemic or local toxicity of commercial drugs, as a result of the integration of the active agents into a variety of biocompatible nanosystems that provide prolonged transport in the bloodstream by reducing interactions with the mononuclear phagocyte system (MPS); (3) High surface/volume ratio of the nanosystems, enabling therapeutic nanoplatforms to undergo effective surface modifications for active and specific tissue targeting. This generates a selective penetration at a molecular scale, allowing a reduction in the dose of use and therefore, a minimization of adverse effects; (4) Controlled release of the therapeutic agent at the target site thanks to external or internal stimuli from the tumour microenvironment (TME) by overcoming the various biological barriers and drug resistance mechanisms.

Despite the considerable technological progress achieved in this area, the main hurdles for nanomedicine to become a new paradigm in cancer therapy derive from: (i) an incomplete understanding of nano–bio surface interactions, (ii) the non-specific targeting of nanosystems, (iii) the heterogeneity of inter- and intratumoural biology, and (iv) the challenges related to the synthesis, manufacturing and control required for clinical translation and subsequent commercialization. Therefore, further studies are still required to improve the high impact of nano-vehicularized drug delivery systems on cancer treatment approaches [2].

With these perspectives, the present review is focused on highlighting the main elements to take into account when nanosystems are used in tumour treatments, with special attention paid to physiological processes as biological barriers or protein corona formation determined by the administration routes. Next, we present the different strategies for nanosystem design proposed in the literature to overcome the hurdles, and the development of promising novel multifunctional nanotherapeutics. Finally, recent clinical trials involving this nanotechnology are revised. 

## 2. Factors Influencing Tumour Treatment with Nanosystems

**Figure 2:**
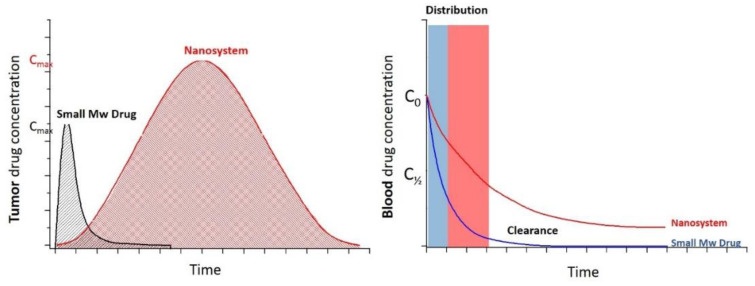
Typical pharmacokinetic profiles of small-molecule drugs and nanosystems in tumours (**left**) and blood (**right**).

Nanosystem-based drug delivery platforms have become an effective strategy to improve low-molecular-weight (LMW) drug pharmacokinetics and the distribution limitations of traditional cancer chemotherapy in physiological environments. Nanosystems have been shown to be advantageous in solubilizing therapeutic loads, substantially prolonging the life of active agents in the circulation. This enables a decrease in the used dose, enhancing the tendency of the drug to accumulate at damaged sites. It also prevents the spreading and free distribution of the drug throughout the body, which would result in undesirable side effects and limit the achievement of the dose required to generate effective responses [8,9]. Figure 2 shows the prolonged circulation properties and enhanced tumour accumulation of nanomedicine therapy versus conventional low-molecular-weight (MW) chemotherapy [10].

The most common route of administration of nanomaterial-based cancer drugs is intravenous injection. This approach avoids the absorption through the intestinal epithelium after oral administration. Even so, there are several factors that severely limit the pharmacokinetics and biodistribution of nanosystems, hindering successful therapeutic outcomes. These factors, described below, have to be taken into account when designing nanoplatforms [8,11,12]. 

### 2.1. Physicochemical Properties of Nanosystems and Protein Corona Formation

The interaction between the nanosystem and the physiological environment will lead to the adsorption of different biomolecules, such as proteins and lipids, on their surface. This process is called “protein corona” formation (PC) [11,12].

**Figure 3:**
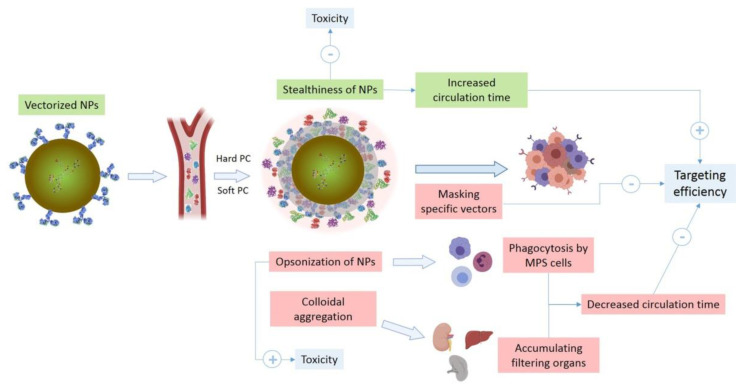
Possible effects on nanoparticle stability, safety and pharmacokinetics due to protein corona formation and interaction after systemic administration.

The opsonisation process that nanoparticles (NPs) undergo after administration into the bloodstream involves the adsorption of plasma proteins, including serum albumin, fibrinogen, fibronectin, apolipoproteins, complement components such as C3b, and immunoglobulins such as IgG or IgM. After this, MPS sequestration will take place. This sequestration is achieved through the binding of these PC proteins to specific receptors on the surface of the phagocytes that are responsible for the removal of pathogens and negatively charged ligands circulating in the blood. This ligand–receptor recognition causes the internalization of NPs, their transport to phagosomes and their fusion with lysosomes [8,12,13] (Figure 3).

This biomolecular interface organization causes the nanosystems to obtain new biological characteristics, which will be presented in Section 3.2. The process of PC formation depends on the competition among the proteins that are to be adsorbed. Miclaus et al. [14] suggested that the development of the nano–bio interface is a dynamic and time-dependent process of exposure of the nanoconstructs (Vroman effect). The proteins with the highest affinity for NP surface chemistry are slowly exchanged, forming a hard corona. In contrast, proteins with low affinity are replaced very quickly, forming a soft corona [15,16,17,18].

The structure of the PC may differ according to the “synthetic identity” of the NPs, i.e., the physicochemical and intrinsic properties of the nanosystems (type of nanomaterial, size, charge, surface functional groups, shape) and the characteristics of the exposure environment (components of the medium, temperature, pH and exposure time) [11,12,15,16,18,19].

The “synthetic identity” will determine the “biological identity” supported mainly by the components of the biocorona. This has implications for NP biological responses, including their pharmacokinetics, biodistribution, membrane adhesion, cell uptake, cell signalling pathways, safety, and therapeutic outcomes [20,21], as detailed in Figure 3. Therefore, it is important to determine the relationship between the physicochemical properties of NPs and their biological activities [15,16,18,22].

Structural changes in proteins bound to NPs are of high biological relevance, since they can cause the loss of protein function, which can give rise to the disruption of physiological homeostasis and to unwanted immune responses. Similarly, massive conformational changes can lead to protein aggregation and to the generation of amyloidal fibres. The interaction of NPs with proteins can induce a variety of signal modulations and toxic effects in biofluids and in cells. Different physicochemical properties of NPs basically contribute to a different corona formation and structural changes in proteins. This conformational change is a dynamic process and depends on the surrounding environment. The reversible or irreversible changes of the protein structure can perturb the downstream signalling, which may consequently be harmful to the host. The formation of the PC is expected to be different in extracellular and intracellular spaces. Once the NPs are internalized (i.e., by receptor-mediated internalization or endocytosis by charge), they can carry toxic consequences directly due to their own chemical characteristics and/or indirectly through the corona formation [23,24].

### 2.2. Processes and Biological Barriers of the Tumour Microenvironment

In order to ensure that anti-tumour treatment based on nanosystems will be effective and have no off-target effects, an efficient supply of the drug is necessary to achieve maximum local activity in the tumour cells. For this reason, it is essential to carry out an exhaustive study of the structure and specific aspects of the TME, which is composed of the extracellular matrix (ECM), the cell system, different biomolecules expressed and secreted by the tumour cells, and the neovasculature.

The TME has a high degree of intra- and inter-tumour heterogeneity, which is a barrier to anti-tumour nanotherapy [12,25,26]. Tumour sites exhibit chaotic ramifications due to a fenestrated and discontinuous vasculature caused by a high production of angiogenic factors, for instance, vascular endothelial growth factor A (VEGF-A), fibroblast growth factor 2 (FGF2), interleukin-8 (IL-8), interleukin-6 (IL-6), hepatocyte growth factor (HGF) or vascular cell adhesion molecule 1 (VCAM-1), among others. Endothelial transcytosis and vascular outbreaks are also present [11,12,26,27].

On the other hand, the ECM is composed of collagen, glycoproteins, glycosaminoglycans, proteoglycans, elastin and hyaluronic acid (HA), among others. It has a structural support function, and provides coupling sites for cell surface receptors such as vascular endothelial growth factor receptor (VEGFR), the integrins αvβ3/5, matrix metalloproteinase receptor (MMPR) or epidermal growth factor receptor (EGFR), among others. In this way, the ECM serves as a reservoir for cytokines and other growth factors [11,25,26,28,29].

In tumour situations, there is an excessive production of ECM and a high density of cells, such as stromalones. Thus, fibroblasts segregate different biomolecules such as collagen type l and remodel the ECM. This gives rise to a lack of perfusion, caused by high interstitial fluid pressure (IFP), poor lymphatic drainage, compression of the blood vessels and solid stress. In addition, this non-controlled growth causes the development of hypoxic regions and a deficiency of nutrients within the tumour (Warburg effect). The hypoxia condition in the tumour results in the inducible hypoxia factor-1α (HIF-1α) being transcribed into the HIF-1α protein, which is deeply associated with the activation of a number of genes that aggravate the tumour condition. All these factors will influence the supply of nanosystems to the target tumour cells [6,11,12,25,26,28,29].

The structural and biochemical characteristics of the tumour microenvironment described above have triggered several interesting and crucial studies about the effective and selective accumulation of nanomedicines in tumours. This has driven researchers to classify the efficient delivery of nanomaterials in the target tissues as either passive or active targeting [25].

### 2.3. Drug Delivery: Passive and Active Targeting

**Figure 4:**
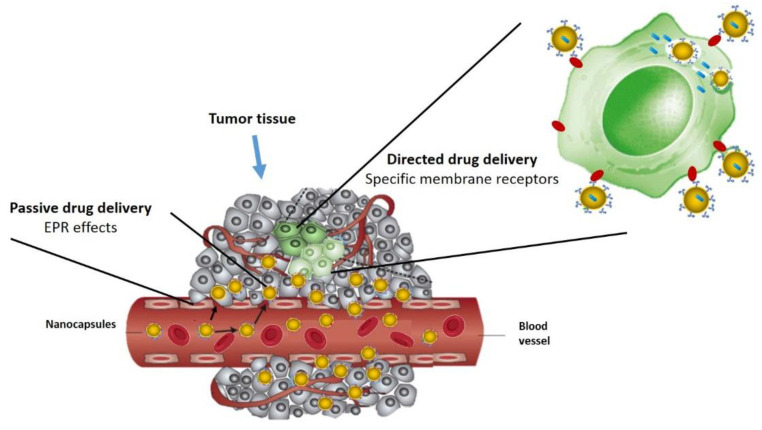
Graphical illustration of passive and active drug targeting. In passive targeting, the nanocarriers pass through the leaky walls and accumulate at the tumour site by the enhanced permeability and retention (EPR) effect. Active targeting can be achieved using specific ligands that bind to the receptors on the tumour cells.

Passive targeting is based on the enhanced permeability and retention effect (EPR), which was reported by Matsumura and Maeda in 1986 [30]. It is related to specific pathophysiological characteristics of tumours versus healthy tissues. This effect reveals the potential of nanomaterials with sizes up to several hundred nanometres for increased passive accumulation. This occurs by extravasation through the fenestrated neovasculature of tumours, since endothelial cells in this type of vasculature have a separation gap from 200 to 2000 nm depending on the tumour type, location and environment. At the same time, the passive accumulation of these nanomaterials is enhanced by the absence of lymphatic drainage in tumour areas, which avoids a quick removal of the nanosystems. This allows the prolonged circulation of the nanomaterial that is required for the administration of the suitable active molecule. Therefore, the EPR effect could be coadjuvant for the targeted administration of biocompatible nanomedicine or macromolecular drugs into the tumour tissue [6,8,9,12]. In order to achieve a successful passive accumulation of nanomaterials mediated by the EPR effect, the physicochemical properties of the nanosystems such as size, morphology, surface charge and surface chemistry must be taken into account. These physicochemical characteristics will establish the pharmacokinetics of the active agents [12]. However, the passive accumulation of nanosystems in the tumour tissue does not avoid their accumulation in other tissues with fenestrated vasculatures such as the liver or the spleen. A deeper tumour penetration is also not guaranteed due to intra- and inter-tumour heterogeneity, which results in a barrier for the development of nanomedicine (Figure 4) [6,8,12]. An approach that still requires further study is the development of individualized nanotherapies [10] that distinguish between tumour phenotypes that are sensitive or not to the EPR effect. The insensitive phenotype has a vasculature with smaller tumour fenestrations, a relatively dense ECM and a more developed immune profile. In contrast, the sensitive phenotype is characterized by a hyper-permeable vasculature with large endothelial fenestrations, a relatively small ECM and a limited immune profile [10,31].

Active targeting is an active orientation or ligand-mediated approach, in which the recognition, retention and uptake of nanosystems rely on their affinity with the target cells. This strategy is based on different specific molecular interactions, such as receptor–ligand interactions, motif-based interactions facilitated by substrate molecules, or charge-based interactions. Surface molecules expressed, present or secreted (proteins, sugars or lipids) by tumour cells in the TME, or even in the surrounding physicochemical environment, compose the target substrates to which nanosystems could be actively directed. For this purpose, biomolecular ligands such as antibodies, proteins, nucleic acids, peptides, carbohydrates, and small organic molecules such as vitamins are incorporated on the surface of the nanoplatforms [12,29]. The rational design of these nanoplatforms has led to the formulation of smart nanosystems with a multivalent nature, which include multiple ligands against the target-specific molecules. This has improved the pharmacokinetics and release of active agents into the tumour and the efficiency of binding molecules, which has generated a greater emphasis on nanotherapy in subsequent studies. However, more research remains to be done in this area, for instance, regarding the stability of the conjugated ligands [12].

As well as the substantial challenges presented by each individual barrier or biological process in TME, it is also important to realise that these will vary in complexity depending on factors such as the route of administration (oral vs. intravenous) and the stage of disease progression (early vs. advanced cancers) [8]. For this reason it is important to take into account the impact that these will have on the final destination of the nanocarriers, as well as the strategies that may be useful for overcoming these obstacles [6].

## 3. Nanosystem Design Strategies for Overcoming the Hurdles

Several nanotechnological strategies make it possible to overcome the barriers mentioned above. These strategies are based on the rational design of nanosystems.

### 3.1. Control of Nanosystem Physicochemical Properties

The physicochemical properties of the nanoparticles, such as size, geometry, surface charge, hydrophobicity and their modification by ligand binding, can be controlled, as described in the following.

Size affects NP clearance and distribution. Large hard particles with diameters above 200 nm are easily accumulated within the spleen and liver, as well as in the capillaries of the lungs due to NP aggregation (especially near the micron range). In contrast, small particles (below 5 nm) are filtered out by the kidneys and, due to their high surface area/volume ratio, can be easily aggregated. It has been established that NPs in the range of 100–200 nm are extravasated through the vascular fenestrations of tumours (EPR effect) and can avoid filtration through the liver and spleen. The current research has shifted to the reduction in NP size, as it has been demonstrated that it decreases the absorption by the MPS [6,11,25,32].

Regarding geometry, different NP shapes exhibit specific flow characteristics. This substantially changes the circulating time life, cell membrane interactions and macrophage uptake of the NP, which in turn influences its bio-distribution among different organs. NPs with traditional spherical geometries present minimal lateral drift and are less likely to migrate to vessel walls and establish points of contact/joint with endothelial cells. Other geometries (cylindrical or discoidal, for instance) are currently being explored thanks to novel top-down and bottom-up manufacturing techniques, and exhibit pronounced effects on pharmacokinetics and bio-distribution. However, spherical geometry is still the most studied [6,11,25].

Controlling the NP surface charge is also necessary. Highly cationic nanosystems are quickly captured by the MPS, in comparison with highly anionic ones. In contrast, neutral or lightly negatively charged NPs show a prolonged circulation half-life, which leads to a better accumulation in the tumours. Thus, the functionalization of nano-transporters with zwitterionic ligands (cysteine and glutathione) or PEGylation has been shown to improve cellular internalization [6,8,11,25]. On the other hand, a greater internalization of positively charged NPs compared with their negatively charged counterparts has been reported for different types of cancer cells, such as HeLa [33,34], MCF-7 [35] and endothelial cells [36]. This has prompted the development of innovative ‘charge conversion’ strategies, which aim to specifically change the charge of NPs in response to environmental stimuli, such as pH. For example, Yuan et al. built doxorubicin-loaded zwitterionic polymer-based NPs, which became positive after extravasation in the acid TME [37]. This facilitated the penetration of the NPs into the tumour cells, improving their response in vivo. The type of surface charge group can also affect targeting. Srivastava et al. analysed the effect of neutral, anionic and cationic charged groups at the surface of carbon NPs on different breast cancer cells at different stages [38]. They showed that, for metastatic phases, anionic phosphate nanoparticles exhibited better targeting activity, while for late phases, sulfonate-functionalized nanoparticles presented higher drug delivery efficiency.

NP hydrophobicity/hydrophilicity establishes its circulation time and thus, the probability of the nanosystems to access the tumoural tissue, since it is known that hydrophilic particles are generally invisible to MPS cells. One of the most frequently used tools to ensure this property is the binding of hydrophilic polymers on the surface [39].

**Figure 5:**
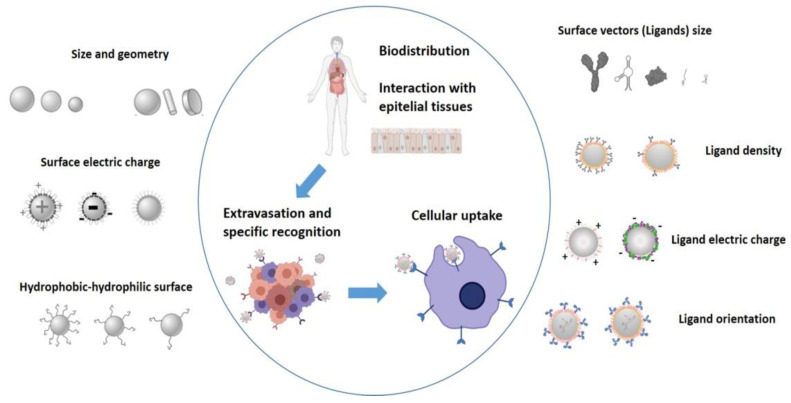
The physicochemical properties of the ligand and the NP affect their blood circulation profiles, biodistribution and their ability to be internalized by cancer cells.

Finally, when conjugating different ligands on the NP surface, the physicochemical properties of the nanosystem (size, geometry and surface properties such as charge) discussed above should be taken into account, as they will affect the functionalization of the bare NPs. At the same time, after functionalization, these same properties will undergo certain changes, affecting bio-distribution and toxicity. The surface charge of the non-functionalized nanosystem will be a decisive property in the conjugation, structure and conformation of the final ligand, since repulsive or attractive forces may appear between the surface of the bare NPs and the functionalizing molecule. Finally, due to cooperative effects, multiple ligands of relatively low affinity can be used to efficiently bind their target. However, this increased affinity is not always linear, and can even increase the density of the ligand, causing harmful effects on cell binding [12]. A schematic representation of the physicochemical properties of ligands and NPs is shown in Figure 5 [40].

### 3.2. Biomimetism

The number of publications focusing on the study of the PC has increased exponentially in recent years. It has been seen that a better understanding of protein adsorption to nanosystems is necessary, since this is going to be decisive for the biological response of NPs, pharmacokinetics, including biodistribution, and, in short, the therapeutic result.

Nanosystems have low stability and high systemic toxicity due to their high surface free energy. Therefore, the formation of PC increases the stability and decreases the toxicity of NPs. However, the formation of the PC through the non-specific binding of the different physiological biomolecules shows disadvantages for in vivo applications. PC can sterically mask ligands, and, as a consequence, NPs can lose their targeting capacity. In addition, the surface ligand can undergo structural changes when interacting with PC proteins, which causes an undesirable bio-distribution and immune response [23,41].

Different strategies to overcome NP opsonization and sequestration by MPS have been defined and are currently being implemented in order to achieve longer circulation times. Several studies have focused on the benefits of PC formation and have suggested approaches to control the biological behaviour of NPs. In this context, two main strategies have been developed: (1) to control the molecular composition of PC and improve biocompatibility, and (2) to prevent PC formation. These two strategies can be carried out by promoting in vivo adsorption with specific proteins (1) or by decorating the surface of nanosystems in vitro with specific proteins (PC) (2), which decreases the absorption of MPS and/or leads preferentially to a targeted delivery [11,23,42].

The influence of the NP physicochemical properties and the medium and experimental conditions on the formation of a protein corona has been also analysed. The main results are summarized in Table 1 and Table 2, respectively.

Different strategies have been carried out to make nanosystems biomimetic:

(1) Functionalization with a hydrophilic and non-ionic polymer such as polyethylene glycol (PEG). The ethylene glycol units build up a close bond with water molecules, making up a hydrophilic layer that greatly protects the NPs from aggregation, phagocytosis, opsonization and subsequent removal by MPS. This leads to an improvement in the stability of NPs in biological fluids, prolonging their circulating lives. Although nanosystems can also be functionalized with materials that have similar protective effects, such as poloxamers (Pluronic^®^), poloxamines (Tetronic^®^), polyvinyl alcohol, poly (amino acids) or polysaccharides, PEG remains the most frequently used. The length and density of PEG determines the targeting ability of the NP, as these properties will mediate the adsorption of plasma proteins. The PEG coating is also frequently used to mask the net positive charge on the surface of nanomaterials, preventing their interaction with negatively charged cell membranes. However, PEG functionalization can lead to immunological problems since non-human pegylated enzymes trigger an anti-PEG antibody-mediated response. This can make it more difficult for nanosystems to be effectively delivered, as they will exhibit a shorter half-life, faster clearance, or prolonged loss of blood circulation. Aggarwal et al. [63] reported that some plasma proteins (e.g., Apolipoprotein E, IgG, etc.) can be adsorbed on PEG-coated surfaces, which shows that this coating does not totally prevent protein adsorption. Muro et al. used hybrid ion surface ligands as an alternative to PEG for reducing PC formation [64]. Strong electrostatic interactions between water molecules and bipolar ligands generate highly stable NP, while minimizing non-specific interactions with biomolecules. Furthermore, Moyano et al. [65] showed that with certain hybrid ion ligands an almost complete inhibition of PC formation occurs in some specific cases, leading to prolonged NP circulation times [38]. For all this, pegylation is still a challenge in the field of nanoformulation [6,8,12,66,67].
(2) In vitro binding of specific proteins to the surface. Ja-Hyoung et al. carried out a pre-coating of NPs with the human epidermal growth factor receptor-type 2 (HER2) protein by combining it with the glutathione-s-transferase construct. They demonstrated that the formation of a protective protein layer can reduce the adsorption of serum proteins while maintaining the selective capacity of the NP [68]. Human serum albumin (HSA) is considered a dysopsonin, i.e., a molecule that prevents sequestration by MPS, promoting a prolonged period of blood circulation. Thiele et al. showed that the adsorption of HSA onto polystyrene microparticles inhibits phagocytosis by dendritic cells [69]. In addition, albumin accumulates in malignant and inflamed tissues and serves as the principal nutrient for tumour growth. Thus, its adsorption favours NP internalization in the target cells [70].
(3) Binding of peptides to NP surfaces. Discher et al. computationally designed CD47 peptides from human CD47, and attached them to the surface of NPs, which prevented NP phagocytosis by macrophages [71].
(4) Coating of NPs with cell membranes. Membranes extracted from autologous leukocytes and red blood cells (RBCs) provide the NP surface with a biomimetic character that substantially prolongs its circulation in vivo [72,73,74]. Tasciotti et al. covered the NPs with leukocyte membranes, achieving approximately a ten-fold decrease in IgG and albumin adsorption onto the NP surface [8,75].

The type of human disease has been also shown to play a critical role in the composition of PC [76]. Thus, the concept of personalized PC has emerged in the field of nanobiotechnology and personalized medicine. Hajipour et al. demonstrated that similar nanoconstructions with PCs formed under incubation with plasma from humans with different diseases offered different responses on the same type of cells [76]. Caracciolo et al. also showed a disease-dependent protein binding and identified relationships between the disease state and the bioidentity of the nanoconstructs [77]. All these findings represent a promising new area in the development of PC for both personalized diagnostics and therapeutic treatments [78].

A biomimetic strategy has to contemplate the NP biological response. Thus, a new challenge in the design of nanosystems is posed by the complexity of establishing relationships between the characteristics of these systems and their biological responses. This is due to the following: (i) The PC is complex and unique for each nanomaterial and nanosystem. Moreover, environments influence PC by interacting with diverse protein compositions through adsorption; (ii) The composition and relative amount of adsorbed proteins may also not correspond to the components of the proteins and the relative concentrations in the exposure media.

Finally, an obstacle for the biomimetic strategy is that current research about the PC structural features at the atomic level is still insufficient to fully evaluate and understand the corona–NP complex. In-depth studies on the structural changes of each PC in NPs would close the gap between NP “applications” and “basic concepts,” as the behaviour of NPs in vivo is definitely related to the orientation and deployment of certain proteins within the corona [23]. Such advances in the field could potentially accelerate the translation of nanotherapeutics into the clinic, as they would ensure the biosafety of the nanotherapeutics [41].

### 3.3. Active Targeting

Several active targeting strategies have been reported to be related to tumour-microenvironment-specific molecular markers [29]:

(1) Using several ECM components: Arg-Gly-Asp peptide (RGD), which recognizes the integrin αvβ3 (transmembrane receptor that works as a tumour-specific marker of angiogenic activity) has been attached to NPs to specifically target angiogenic vessels, increasing their specific accumulation at the tumour site, and thus reducing toxicity [79]. Functionalization with ligands that target EGFR is carried out with monoclonal antibodies, fragments (cetuximab, trastuzumab or panitumumab) or endogenous ligands such as epidermal growth factor (EGF). This potentially increases the EPR effect in different tumour types, as demonstrated by Zalba et al. [80]. They developed EGF-conjugated liposomes targeted against EGFR, causing a significant decrease in IC_50_ oxaliplatin in EGFR-positive colorectal cancer cell lines [29]. Other nanocarriers have been designed to inhibit the expression of matrix metalloproteinases (MMPs), considered as tumour biomarkers. They integrate MMP substrates (collagen, gelatin, fibrinogen, etc.). Synthetic substrates sensitive to MMP are easy to incorporate and, at the same time, offer selectivity and sensitivity. However, the responsiveness of NPs to MMP varies with the peptides used [32,81]. Functionalization with HA (the main component of ECM) leads to the targeting of the residual sugar of CD44 receptors [82], which has an increased expression in a large number of tumours. Liu et al. demonstrated that HA-protected NPs of 200 nm showed an optimal EPR effect in mice with 4T1 mouse breast tumours [83]. This provided a dramatic suppression of primary tumour growth (95%), and an important inhibition of tumour metastasis (90%). Furthermore, multifunctional dual-orientation nanosystems with EGFR and CD44 have been recently developed for the improvement of the EPR effect. Although the dual combination of EGFR and HA has not been widely studied yet, it has appeared as an effective anti-tumoural nanotherapy to reduce the uncertainty of the single target [84,85].
(2) Using tumour-specific pathophysiological conditions, such as the hypoxia. Small interfering RNA (siRNA) has been administered and vectored against HIF-1α [86]. In another study, NPs were functionalized with Saposin C, a lysosomal protein that binds to phosphatidyl serine (a specific molecular marker of hypoxia and apoptosis) of hypoxic TME. The resulting NPs showed remarkable therapeutic efficacy in the tumour model, crossing the blood–brain barrier, exhibiting specific retention in tumour tissue, and sensitizing hypoxic cells [87].

### 3.4. Adaptive Nanomedicine

The adaptive nanomedicine aims to improve access to the tumour by directly remodelling or modifying blood vessels, stroma and cancer cells through different mechanisms or agents [31]. This strategy is especially important when the tumour phenotype is insensitive to the EPR effect. Some examples are presented in the following:

(1) Vascular agents such as bradykinin, serotonin, histamine, prostaglandins, nitrous oxide or tumour necrosis factor α (TNFα) induce a state similar to inflammation. This improves the vascular permeability of the therapeutic NP. All these vasomodulators are toxic if they are administered freely. However, such a toxicity is drastically reduced when they are encapsulated in nanosystems. Another strategy is the remodelling of the tumour vasculature. Jain et al. investigated the administration of antiangiogenic or angiogenic agents such as VEGF [88,89] and the functionalization of nanosystems with anti-VEGF receptor antibody [90]. This improved the transvascular delivery of NPs in murine tumour models [8,28,31].
(2) Enzymatic agents, such as collagenase [91] and hyaluronidase [92,93], digest the physical structure of the ECM, reducing IFP in tumours, while direct induction of the apoptosis of TME cells can reduce pressure on the microvasculature in order to improve the depth of penetration of the nanosystems in the tumours [31]. Jain et al. used losartan, an angiotensin II receptor antagonist and anti-fibrotic agent that decompresses tumour vessels, increases tumour perfusion, and inhibits the synthesis of collagen I by fibroblasts associated with carcinoma. This improved the accumulation and efficiency of liposomes containing doxorubicin [94]. On the other hand, Chen et al. investigated a synergistic enzyme therapy that altered the tumour vasculature. Nanocarriers containing MMP-9-activatable doxorubicin (DOX) prodrug were combined with nanocarriers loaded with combetastatin A4 (CA4, carbonic anhydrase 4). CA4-loaded nanosystems altered tumour blood vasculature and selectively enhanced MMP-9 expression in tumours to promote doxorubicin accumulation, leading to the effective treatment of 4T1 and C26 tumours [95].
(3) The use of external stimuli to physically modify a delivery site (by reconfiguring TME, increasing vessel leakage or destroying physical barriers in the TME) before or simultaneously with the delivery of nanosystems enhances the EPR effect. This includes radiation, ultrasound, hyperthermia and photodynamic therapy [10,29,31,96].

### 3.5. Complementary Diagnosis of the EPR Effect

Complementary diagnosis consists of the stratification of patients based on their tumour characteristics in order to establish if the patients have a tumour phenotype that is sensitive to the EPR effect [8]. Several strategies based on the use of biomarker profiles and image data are currently being investigated. The first strategy aims to identify circulating proteins associated with the TME and positively correlated with the EPR effect [11]. Yokoi et al. demonstrated that the ratio of matrix metalloproteinase 9 (MMP-9) to tissue inhibitor metalloproteinase 1 (TIMP-1) was indicative of vascular permeability, since MMP-9 degrades the dense collagen matrix of the basement membrane. These findings suggest that these metalloproteinases can be used as potential biomarkers for EPR and a way to identify patients who would benefit most from the administration of NPs. These authors also pointed out a direct correlation between the content of collagen in normal vasculature and the delivery of NPs, suggesting another potential marker for patient stratification [8,97]. Moreover, image-based nanodiagnostics can be performed to monitor the bio-distribution and accumulation of nanomedicines at the target site. This would be achieved by incorporating contrast agents (e.g., pherumoxitol) into therapeutic NPs and by using protocols for imaging blood vessels and EMT, such as photon emission computed tomography (SPECT) or magnetic resonance imaging (MRI). However, imaging of the EPR effect is still in the developmental stage [2,10,11]. The final purpose is the selection of patients who have a higher probability of a positive response to a therapeutic treatment based on the EPR effect. However, to achieve real usefulness in clinics, these approaches should be further validated by means of precise correlative studies, defining a clear set of parameters and criteria capable of predicting the therapeutic outcome [11].

## 4. Novel Multifunctional Nanotherapeutics

Nanomedicine against cancer must be supported by the rational, biomimetic and systematic design of optimal therapeutic combinations. In recent decades, there has been an exponential increase in the interest in theranostic nanoplatforms. These nanosystems combine imaging agents, payload drugs, and targeting residues to control treatment effects and to monitor therapeutic efficacy. All diseases could benefit from the rapid diagnosis and treatment achieved with theranostics. However, due to the high morbidity rate of cancer, initial research on theranostics has focused on oncology [25,98,99].

Many smart nanosystems with diagnostic and therapeutic functions have been designed to be activated by/on TME, and they are considered an effective strategy to improve sensitivity and theranostic selectivity. Diagnostic and therapeutic functions could only be activated at the target site by special exogenous stimuli (e.g., light, magnetism, ultrasound) or endogenous stimuli that correspond to parameters specific to the TME (e.g., pH, redox, ATP, H₂O₂, glutathione (GSH), hypoxia, mechanical strength, and enzymes). In addition, obtaining diagnostic signals activated by/on TME provides useful information regarding the change in physiological parameters of the tumour cells and can alter the therapeutic strategy in real time (monitoring) [96,100,101].

Currently, the design of transformable nanosystems or nanosystems synergically combined with multitherapeutic functions is becoming very important. They can elude the multiple obstacles or biological processes to improve the delivery efficiency and, therefore, to achieve a complete elimination of the tumour. Xue et al. developed a nanosystem with a charge and size that can be changed for the targeted delivery of nanotherapies. This nanosystem is composed of amphiphilic monomers obtained by the conjugation of two components: a hydrophobic photosensitizer with intrinsic magnetic resonance capabilities (Pa), and a hydrophilic antineoplastic drug (DOX). These components are bound through hydrazone bonds that can be excised at intracellular acidic pH. The binding of PEG on the surface reduces the positive surface charge, which allows an improvement in the blood circulation time. In the acid TME, PEG cleavage occurs, causing a change in the nanosystem size and surface charge. This significantly improves penetration into the tumour tissue and general cell internalization. At the same time, the presence of the contrast agent (Pa) provides a visualization of the in vivo distribution in real time and the therapeutic efficacy in a non-invasive way [102,103].

Multi-drug resistance is caused by the overexpression of drug flow transporters, such as Permeability glycoprotein (P-gp), ATP-binding Cassette super-family G member 2 (ABCG2) or super-family B member 1 (ABCB1), and breast-cancer-resistant protein (BCRP). MDR is a significant obstacle in the prognosis and treatment of breast cancer [68]. To overcome this limitation, it is necessary to elude the recognition of the transporter, and inhibit its expression or its function. Different nano-based strategies have been developed against MDR [104]. Deng et al. [105] designed doxorubicin-loaded NPs by generating a film that alternated small interfering RNA (siRNA) with poly-lactic acid (PLA), coated with HA molecules. Patients with triple-negative breast cancer are usually non-responsive to doxorubicin treatment, however, this nanomedicine inhibits the expression of the MDR-associated protein gene, through the delivery of siRNA. It has been shown that many patients treated with trastuzumab develop primary or secondary resistance [106]. Thus, different nanosystem formulations against breast cancer have emerged to reduce this type of resistance: (1) The combined treatment of IgG-HER2 and DOX produces a synergistic anti-tumour effect against breast tumour cells that overexpress HER2 [107]. Although DOX may cause cardiac toxicity, IgG-HER2-functionalized DOX-loaded nanosystems enhance the anti-tumour effect, reducing such cardiotoxicity [103]. Osuna et al. designed Protein A-functionalized immunostimulating complexes (ISCOM) coupled with trastuzumab and loaded with paclitaxel or doxorubicin [106]. They obtained a greater accumulation of the nanosystem towards the cell lines that overexpress HER2 regardless of antibody resistance. (2) Chiang et al. designed nanocapsules with poly-(vinyl alcohol) (PVA) and a pH-sensitive polymer functionalized with a thiol group (PMAA). They also incorporated iron oxide for magnetic orientation. In this nanoconstruction they simultaneously encapsulated paclitaxel (hydrophobic) and doxorubicin (hydrophilic), functionalized with trastuzumab [108]. This double-duality strategy showed cell death in vitro and the suppression of tumour growth in vivo, which provides multiple benefits in cancer therapy. 

Finally, the power to hold diagnostic, therapeutic and monitoring agents in the same nanocarrier, with properties that provide controlled release at the tumour site, opens up new possibilities in anti-tumour research.

## 5. Clinical Trials

In recent decades, progress has been made in the fight against cancer due to the successful implementation of several novel nanomedicine products in clinical trials and even in the market [109]. However, the clinical translation of nanomedicines still remains a challenge, as it requires a detailed understanding of the physicochemical properties, internal and external structure, chemical reactivity and stability, bio-distribution, toxicity and bio-compatibility of nanosystems, among others. Even with these and other challenges, some first-generation nanomedicines have already been recognized in the clinical cancer research community as effective medical tools. For instance, Doxil, a pegylated liposomal formulation of doxorubicin for the therapy of different cancer types, was approved by the FDA in the mid-1990s. Doxil exhibited reduced cardiotoxicity in comparison to free doxorubicin, but with the same therapeutic efficacy. Additionally, it enhanced pharmacokinetic parameters, with an important reduction in drug clearance rate and a 20-fold increase in bioavailability. This allowed a better biodistribution than other formulations (non-pegylated liposomes) or free doxorubicin [110,111]. Abraxane, a nanoscale albumin-bound form of paclitaxel, was also approved by the FDA in 2005 [112]. Table 3 collects some clinically approved nanoparticle-based cancer therapeutics.

Nowadays, there are numerous nanosystems in clinical trials that have not yet been approved by the FDA or other regulatory agencies [113,114]. Table 4 collects some of them. The majority of nanosystems in clinical/commercial development are therapeutic, and most of them are organic, in order to achieve relatively low toxicity and high biocompatibility. However, without a doubt, one of the recurring strategies in the design of many nanoformulations seeks a specific surface functionalization that minimizes the opsonization process using different hydrophilic polymers, such as PEG, pluronics or polysaccharides. On the other hand, one of the few approved cancer imaging nanomedicines is the superparamagnetic iron oxide nanoparticle (SPIO) for magnetic resonance imaging. While there are still some in clinical trials or experimental study stages, some SPIO products have been approved by various regulatory agencies, including the USA FDA. The most promising products for cancer diagnosis are Resovist, Endorem, and Feridex [115].

A novel method related to diagnostic medicine has been developed to detect prostate cancer circulating tumour cells. It is based on the biological epithelial–mesenchymal transition by a near infrared emissive nanotechnology device (NCT02022904). This clinical trial was started in June 2015 and its objective is to determine whether circulating tumour cells can be captured using the novel mesenchymal-marker-based near-infrared-emissive polymersomes.

In another clinical trial (NCT03656835), a pilot study of nanochip technology is being developed. It is a collaboration, started in 2018, between the Ohio State University Comprehensive Cancer Center and the National Cancer Institute. This trial explores the efficacy of a biochip of immunogenated lipoplex nanoparticles in the monitoring of the response to treatment and the detection of recurrences in participants with diffuse large B-cell lymphoma.

In order to further expand the potential of nanoparticles for simultaneous imaging and therapeutic applications, researchers have increasingly started to explore various inorganic matrices, such as gold, iron oxide, quantum dots, and silica nanoparticles. In this way, nanomedicines can be developed as theranostics, which have both imaging and therapeutic potential. However, the clinical development of theranostic agents is still preliminary and presents several challenges.

## 6. Conclusions

There are still many hurdles in the development of nanosystems against cancer, many of which have been presented in this paper. However, thanks to the basic research focused on these areas, we are getting closer to solving them by approaching potential tools for the treatment and management of cancer, obtaining “real” medical solutions based on nanomedicine. As nanotechnology offers completely new alternatives that have not been achieved by conventional approaches until now, many limitations have been overcome by multidisciplinary research.

Among the potential tools developed in recent decades that have led to an effective transformation in the field of nanomedicine against cancer, we find the design of smart and/or theranostic nanomaterials. They deliver therapeutic payloads in response to various stimuli, which provides an attractive multifunctionality with highly relevant properties such as specificity, sensitivity and selectivity. This is achieved through a rigorous control over the nanosystem physicochemical properties and a compression of the interactions of the nano–bio interface. Moreover, the early detection of tumours, non-invasive monitoring after the administration of a therapeutic nanosystem, and the evaluation of therapy outcome are key factors for successful anti-tumour treatment. This improves the survival rate, while minimizing the risks and costs compared with conventional treatment. However, the clinical translation of these nanomedicines remains a difficult task. Two vital issues urgently need to be solved: firstly, assessing the biosafety of these platforms by systematically evaluating the toxicity and in vivo behaviour of nano-formulations; and secondly, the achievement of the expected effect in the clinic.

## Figures and Tables

**Table 1:** Effect of the NP physicochemical properties onthe generation of the Protein Corona. Adapted from [20].

NP Property	Effect on the Protein Corona	References
Size	Well-ordered hard corona and thin soft corona in larger NPs.	[43]
	Less perfect packing of hard corona and random soft corona in smaller NPs. In small NPs, proteins do not undergo drastic structural modifications.	[44]
Shape	Shapes exhibiting higher surface area absorb more protein (stars more than rods)	[45]
Hydrophilicity/ hydrophobicity	Hydrophobic NPs attract hydrophobic proteins by hydrophobic interaction.	[46]
	Hydrophobic NPs attract hydrophobic domains of proteins, favouring protein denaturation/conformational changes.	
	Hydrophilic NPs interact with more polar and charged proteins through electrostatic interactions.	
Surface charge	NPs with high density of charge tend to form thicker and denser PCs.	[47]
	Highly positively charged NPs interact very quickly and very strongly with proteins with IP (immunoprecipitation) <5.5. Highly negatively charged NPs interact mostly with proteins with IP >5.5. Slightly negatively charged NPs presentfewer interactions with proteins.	[48]

**Table 2:** Medium and experimental conditions influencing the generation of the Protein Corona. Adapted from [20].

Medium/Experimental Conditions		Effect on the Protein Corona	References
Medium	Protein amount	The protein amount forming the PC depends on the serum type and concentration.	[48,49]
	Composition	PC composition depends on the biofluid origin (e.g., interstitial fluid, blood, plasma, serum). The change in extracellular medium during circulation affects PC composition.	[50,51,52,53]
	Source	In human samples, inter-individual variability (age, diet and health state) has been shown to influence PCs.	[54,55]
Temperature and pH		Temperature and pH influences the protein diffusivity and the electrostatic interaction NP-protein.	[56,57]
		Protein structural stability is affected by plasma temperature and pH resulting in an exchange of proteins from PC. Temperature and pH modifications do not significantly modify the PC abundance of proteins with high affinity towards NPs.	
Time		PC is formed rapidly around NPs (<0.5 min), and over time, although the total amount and composition of the PC do not change significantly, the abundance of each protein can fluctuate.	[58,59]
Fluidics		Dynamic conditions drive to an in vivo PC molecularly richer in comparison to its counterpart ex vivo PC, although the total amount of protein attached to NPs is similar to that from in vitro conditions.	[60,61,62]
		PC conformation is more heterogeneous upon dynamic conditions, leaving uncoated NP moieties free to interact with cells.	

**Table 3:** Clinically approved nanoparticle-based cancer therapeutics.

Composition		Trade Name	Company	Indication	Administration *
Liposomal platforms	Liposomal doxorubicin	Myocet	Zeneus, England, UK	Combination therapy with cyclophosphamide in metastatic breast cancer	i.v.
	Liposome-PEG doxorubicin	Doxil/ Caelyx	Ortho Biotech, Schering-Plough, NJ, USA	HIV-related Kaposi’s sarcoma, metastatic breast cancer, and metastatic ovarian cancer	i.m.
Polymeric platforms	Methoxy-PEG-poly(D,L-lactide) Taxol	Genexol-PM	Samyang, Seoul, Korea	Metastatic breast cancer	i.v.
	PEG–L-asparaginase	Oncaspar	Enzon, NJ, USA	Acute lymphoblastic leukaemia	i.v. i.m.
Other platforms	Albumin-bound paclitaxel	Abraxane	Abraxis BioScience, AstraZeneca, LA, USA	Metastatic breast cancer	i.v.

* i.v.: intravenous administration, i.m.: intramuscular administration.

**Table 4:** Nanoparticle-based cancer therapeutics in clinical trials.

Composition		Trade Name	Company	Indication	Administration *	Status
Liposomal platforms	Liposomal annamycin	L-Annamycin	Callisto, NY, USA	Acute lymphocytic leukaemia, acute myeloid leukaemia	i.v.	Phase I
	Liposomal cisplatin	SLIT Cisplatin	Transave, NJ, USA	Progressive osteogenic sarcoma metastatic to the lung	Aerosol	Phase II
	Liposomal doxorubicin	Sarcodoxome	GP-Pharm, Barcelona, Spain	Soft tissue sarcoma	i.v.	Phase I/II
	Liposomal lurtotecan	OSI-211	OSI Pharmaceuticals, NY, USA	Ovarian cancer	i.v.	Phase II
	Liposomal vincristine	Onco TCS	Inex, Enzon, NJ, USA	Non-Hodgkin’s lymphoma	i.v.	Phase II/III
Polymeric platforms	HPMA copolymer–DACHPlatinate	ProLindac	Access Pharmaceuticals, TX, USA	Ovarian cancers	i.v.	Phase II
	PEG–arginine deaminase	Hepacid	Phoenix, Mannheim, Germany	Hepatocellular carcinoma	i.v.	Phase I/II
	PEG–camptothecin	Prothecan	Enzon, NJ, USA	Various cancers	i.v	Phase I/II
	Pluronic block-copolymer Doxorubicin	SP1049C	Supratek Pharma, QC, Canada	Oesophageal carcinoma	i.v.	Phase II
	Polycyclodextrin camptothecin	IT-101	Insert Therapeutics	Metastatic solid tumours	i.v.	Phase I
	Polyglutamate camptothecin	CT-2106	Cell Therapeutics, LA, USA	Colorectal and ovarian Cancers	i.v.	Phase I/II
	Polyglutamate paclitaxel	Xyotax	Cell Therapeutics, WS, USA	Non-small-cell lung cancer, ovarian cancer	i.v.	Phase III
	Poly(iso-hexyl-cyanoacrylate) Doxorubicin	Transdrug	BioAlliance Pharma, Paris, France	Hepatocellular carcinoma	i.a.	Phase I/II
Other platforms	Nanocrystalline 2-methoxyestradiol	Panzem NCD	Elan, EntreMed, NY, USA	Various cancers	Oral	Phase II
	Paclitaxel nanoparticles in porous, hydrophilic matrix	AI-850	Acusphere, MA, USA	Solid tumours	i.v.	Phase I

* i.v.: intravenous administration, i.a.: intraarterial administration.

## Data Availability

Not applicable.
